# Ensuring safety of exercise training through non‐invasive measurement of cardiac function: A pilot study in adults

**DOI:** 10.14814/phy2.70768

**Published:** 2026-02-24

**Authors:** Takayuki Fujiwara, Eisuke Amiya, Masao Takahashi, Atsuko Nakayama, Yuto Konishi, Masanobu Taya, Kanako Hyodo, Naoko Takayama, Issei Komuro, Norihiko Takeda

**Affiliations:** ^1^ Department of Cardiovascular Medicine The University of Tokyo Hospital Bunkyo‐ku Tokyo Japan; ^2^ Department of Computational Diagnostic Radiology and Preventive Medicine The University of Tokyo Hospital Bunkyo‐ku Tokyo Japan; ^3^ Department of Cardiovascular Medicine JR Tokyo General Hospital Shibuya‐ku Tokyo Japan; ^4^ Department of Cardiology Sakakibara Heart Institute Fuchu Tokyo Japan; ^5^ Department of Rehabilitation The University of Tokyo Hospital Bunkyo‐ku Tokyo Japan; ^6^ Department of Frontier Cardiovascular Science The University of Tokyo Hospital Bunkyo‐ku Tokyo Japan; ^7^ International University of Health and Welfare Minato‐ku Tokyo Japan

**Keywords:** aerobic exercise training, AESCULON, brain natriuretic peptide, cardiopulmonary exercise testing, exercise capacity

## Abstract

Cardiac rehabilitation (CR) improves exercise capacity, but frequent cardiopulmonary exercise testing (CPET) is impractical. The AESCULON mini enables non‐invasive hemodynamic monitoring, though its role in CR remains unclear. Eleven patients (6 myocardial infarction, 3 angina pectoris, 2 dilated cardiomyopathy) undergoing outpatient CR at the University of Tokyo Hospital were studied. Hemodynamics were measured using the AESCULON mini before and after 20 min of aerobic exercise at the anaerobic threshold. CPET and brain natriuretic peptide (BNP) were assessed within 2 weeks. Stroke volume, cardiac output, and cardiac index tended to increase, and thoracic fluid content (TFC) decreased post‐exercise. TFC before (*r* = 0.767, *p* = 0.006) and after (*r* = 0.711, *p* = 0.014) correlated with BNP. Changes in stroke volume and cardiac output correlated with peak VO_2_, percent predicted peak VO_2_, and ΔVO_2_/ΔWR. Patients with increased cardiac output during exercise had higher peak VO_2_ and ΔVO_2_/ΔWR. Non‐invasive hemodynamic data from the AESCULON mini correlated with BNP and exercise capacity, suggesting its usefulness for detecting heart failure progression and estimating exercise capacity in CR.

## 1 INTRODUCTION

Cardiac rehabilitation (CR) is a well‐established intervention for improving cardiopulmonary exercise capacity and preventing recurrence of cardiovascular diseases (Taylor et al., 2022). The components of CR include evaluation of the patient's condition, exercise prescription, physical activity counseling, exercise training, interventions for cardiovascular risk factors, nutritional counseling, and psychosocial support (Brown et al., 2024). The purpose and methods of CR vary according to the disease phase. In the acute phase, CR is typically conducted in the hospital ward to help patients regain activities of daily living, with bedside mobilization performed alongside acute treatment to prevent frailty. Education programs for disease management are also provided.

In the recovery phase, when patients are ready to return to daily life, supervised exercise training begins in the CR room. Aerobic exercise capacity is usually evaluated by cardiopulmonary exercise testing (CPET) to determine exercise prescription (Herdy et al., 2016), unless patients are unable to undergo CPET due to low physical fitness or impaired cardiopulmonary function. Exercise intensity for aerobic training is determined using parameters such as peak VO_2_, heart rate reserve, and the anaerobic threshold (AT) (Hansen et al., 2022). However, optimal training intensity may vary over time with the patient's condition, and frequent CPET is often impractical due to its resource‐intensive nature.

Recently, non‐invasive medical devices capable of measuring hemodynamic parameters have been developed (Nguyen & Squara, 2017) and there are several reports which described the accuracy and usefulness of non‐invasive monitoring of hemodynamics using AESCULON mini monitor in adults (Chen et al., 2018; Nakayama, Nakao, et al. 2020; Nakayama, Iwama, et al., 2020; Nguyen & Squara, 2017; Trinkmann et al., 2016) and children (Tirotta et al., 2017; Yoshitake et al., 2017). This study aimed to evaluate the relationship between hemodynamic parameters measured by the AESCULON mini before and after aerobic exercise, and exercise capacities assessed by CPET or brain natriuretic peptide (BNP) levels, to explore how non‐invasive monitoring could be applied to CR.

## 2 METHODS

### 2.1 Patients

This study enrolled 11 patients who were referred to the outpatient cardiac rehabilitation department in the University of Tokyo Hospital between September 2015 and December 2016. All components of standard informed consent, including the purpose of the study, risk, and benefits, were fully explained to the patients before enrollment. Written informed consent was obtained from each patient. The study protocol conformed to the tenets of the Declaration of Helsinki and was reviewed and approved by the Institutional Review Board of the University of Tokyo, Tokyo, Japan (10760).

### 2.2 Data collection

BNP level was assessed using the standard laboratory method at the University of Tokyo Hospital within 2 weeks before or after the non‐invasive measurement of cardiac functions by AESCULON mini (Osypka Medical, Berlin, Germany). For the assessment of exercise capacity, cardiopulmonary exercise testing (CPET) was performed within 2 weeks before the non‐invasive measurement of cardiac functions by AESCULON mini.

### 2.3 Exercise training and non‐invasive measurement of cardiac functions using AESCULON mini monitor

Patients performed a 20‐min session on a bicycle ergometer, with work load prescribed according to AT determined by CPET. Thoracic electrical bioimpedance (TEB) measurement was performed by the AESCULON mini monitor before and after exercise training. The AESCULON mini monitor emitted a high frequency (50 kHz) and low‐amperage (2 mA) alternating electrical current of constant amplitude via a pair of surface electrodes across the left side of the thorax (Schmidt et al., 2005). Before aerobic exercise, ECG surface electrodes were attached side to side in a vertical direction to the patients left middle and lower neck, and to the lower thorax at the left mid‐axillary line, at the level of the heart and xiphoid process. Then, the electrodes were connected to the AESCULON mini monitor. Hemodynamic parameters including stroke volume (SV), stroke index (SI), cardiac output (CO), cardiac index (CI), and thoracic fluid content (TFC) were measured in a sitting position at an bicycle ergometer before and within 3 min after exercise as previously described (Tomaske et al., 2008). Based on the principle of electrical velocimetry, SV is derived from changes in thoracic electrical bioimpedance that reflect aortic blood flow dynamics. SV was obtained by the multiplication of volume of electrically participating tissue calculated as a function of body weight and height (V_EPT_), ohmic equivalent of mean aortic blood velocity during left ventricular ejection ($\bar{\nu }$_LVET_), flow time derived from left ventricular ejection time normalized for RR interval (FT_c_).

$$\mathrm{SV}=V_{\mathrm{EPT}}\times {\bar{\nu }}_{\text{LVET}}\times {\text{FT}}_{c}$$

The ohmic equivalent of mean blood flow acceleration in the ascending aorta was calculated by the maximum rate of change of TEB over that area of the thorax, and the AESCULON mini monitor incorporates an algorithm which transforms the ohmic equivalent of mean aortic blood flow acceleration into an equivalent of mean aortic blood flow velocity.

$${\bar{\nu }}_{\text{LVET}}={\left[\frac{{\left(\mathrm{dZ}\left(t\right)/\left(\mathrm{dt}\right)\right)}_{\max}}{Z_{0}}\right]}^{n}$$

[(dZ(t)/dt)_max_] is the maximum rate of change of TEB during systole. *Z*_*0*_ is the base impedance (average value over 10 cardiac cycles) and *n* is an exponent which is <1 (Schmidt et al., 2005).

CO was calculated as the product of SV and heart rate. SI and CI were calculated as the ratio of SV and CO to body surface area, respectively. TFC is derived from the TEB impedance (1/base impedance) (Sumbel et al., 2020). The Reproducibility of CO measurement by AESCULON was reported to be high (bias 0 ± 8%, percentage error 15%) (Trinkmann et al., 2016).

### 2.4 Statistical analysis

Continuous variables are presented as mean ± SD. Paired *t*‐tests were used to compare pre‐ and post‐exercise parameters. Pearson's correlation coefficients assessed relationships between AESCULON‐derived data and CPET or BNP values. The analyses were performed using SPSS 18.0/Windows SPSS, Inc., (Chicago, IL, USA) statistical software. A *p* value <0.05 was considered statistically significant.

## 3 RESULTS

**FIGURE 1:**
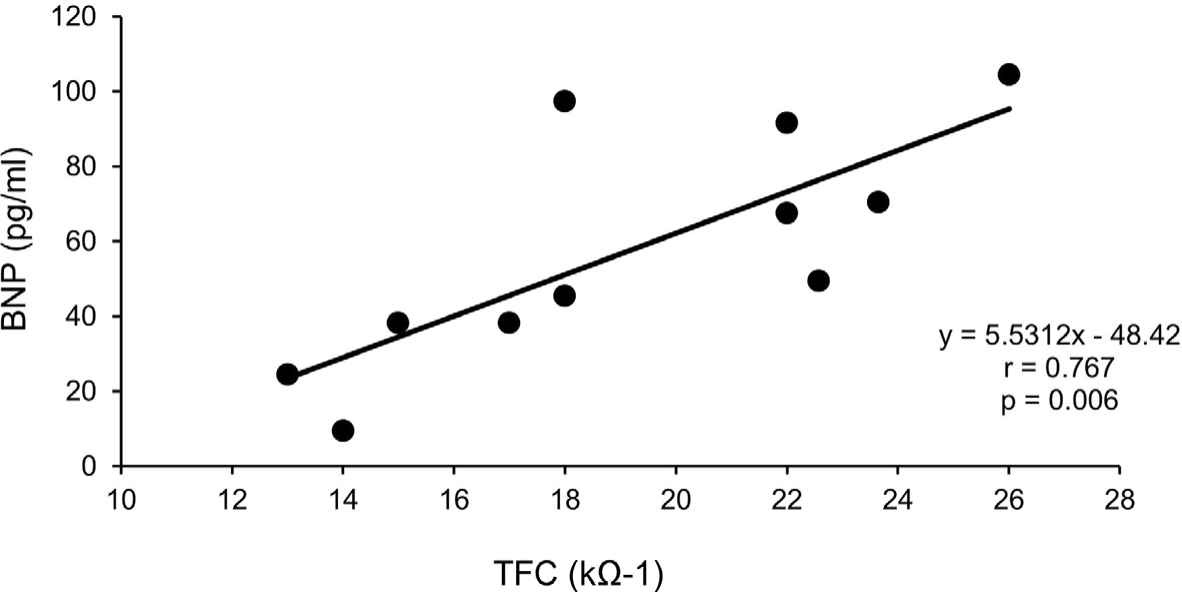
Correlation between BNP and TFC measured before aerobic exercise. BNP, brain natriuretic peptide; TFC, thoracic fluid content.

**FIGURE 2:**
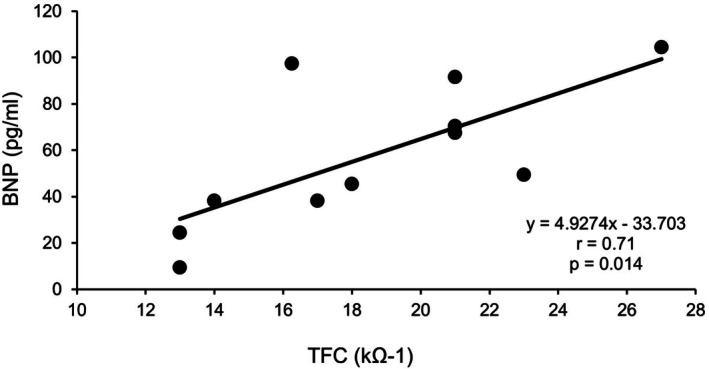
Correlation between BNP and TFC measured after aerobic exercise. BNP, brain natriuretic peptide; TFC, thoracic fluid content.

Between September 2015 and December 2016, 11 patients participated in the study. Six had myocardial infarction, three had angina pectoris, and two had dilated cardiomyopathy. Patient characteristics, including BNP levels and CPET results, are shown in Table 1. Average level of peak respiratory exchange ratio (RER) was 1.12 and RER was greater than 1.00 in all patients. There were six patients (54.5% of all) with use of beta blocker, but there was no significant difference in percent predicted heart rate during CPET between the patient with beta blocker or not (70.4 ± 14.8 vs. 74.4 ± 12.3, *p* = 0.639). Hemodynamic parameters (SV, SI, CO, CI) tended to increase following aerobic exercise at the AT level, while TFC tended to decrease; however, none of these changes reached statistical significance (Table 2). We evaluated the correlation between the exercise capacities assessed by CPET or levels of BNP, and hemodynamic parameter obtained by AESCULON mini monitor before and after aerobic exercise. A significant positive correlation was observed between TFC values of both pre (*r* = 0.767, *p* = 0.006) (Figure 1) and post exercise (*r* = 0.711, *p* = 0.014) (Figure 2), and BNP levels.

**TABLE 1:** Patient characteristics.

	Whole group (*n* = 11)
Sex, male/female (%)	7/11 (63.6)
Age, years	61.5 ± 11.7
Height, m	1.65 ± 0.09
Weight, kg	61.5 ± 11.7
BMI, kg/m^2^	22.3 ± 2.84
BSA, m^2^	1.67 ± 0.19
MI/AP/DCM, *n* (%)	6/3/2 (54.5/27.3/18.2)
BNP (pg/mL)	57.8 ± 31.3
Peak VO_2_ (mL/kg/min)	15.2 ± 2.72
VO_2_ at AT (mL/kg/min)	11.2 ± 1.36
ΔVO_2_/ΔWR (mL/min/watt)	7.88 ± 2.2
VE/VCO_2_ slope	30 ± 5.71
Minimum VE/ VCO_2_ (mL/mL)	31.5 ± 7.47
Peak RER	1.12 ± 0.07

*Note*: Continuous variables as mean ± SD.

Abbreviations: AP, angina pectoris; AT, aerobic threshold; BNP, brain natriuretic peptide; BMI, body mass index; BSA, body surface area; DCM, dilated cardiomyopathy; MI, myocardial infarction; RER, respiratory exchange ratio; VE, minute ventilation; WR, work rate.

**TABLE 2:** AESCULON mini hemodynamic data before and after exercise at AT level.

Hemodynamic parameters	Before	After	*p* value	ΔAfter‐before
SV (mL)	57.7 ± 7.51	59.9 ± 5.69	0.214	2.21 ± 6.01
SI (mL/m^2^)	34.9 ± 5.71	36.1 ± 4.00	0.265	1.21 ± 3.68
CO (l/min)	3.78 ± 0.84	4.01 ± 0.87	0.102	0.23 ± 0.44
CI (l/min/m^2^)	2.28 ± 0.58	2.41 ± 0.55	0.128	0.13 ± 0.27
TFC (1/Ω)	19.2 ± 4.62	18.6 ± 4.66	0.071	−0.63 ± 1.03

*Note*: Continuous variables as mean ± SD.

Abbreviations: CI, cardiac index; CO, cardiac output; SI, stroke index; SV, stroke volume; TFC, thoracic fluid content.

**FIGURE 3:**
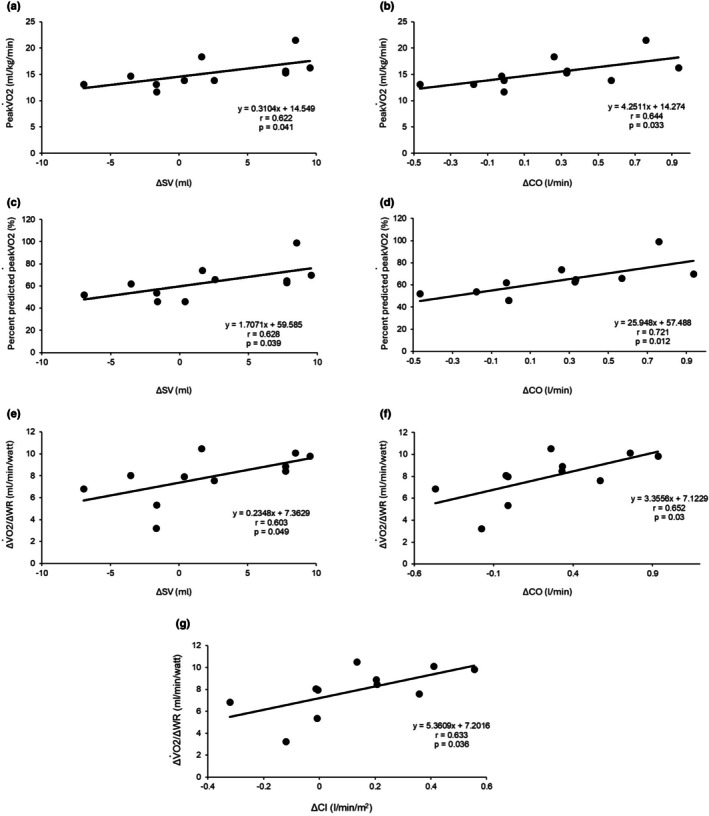
Correlation between exercise capacities obtained by CPET and changes in hemodynamic parameters during aerobic exercise training measured by AESCULON mini. Correlation between peak VO_2_ and changes in SV (a), peak VO_2_ and changes in CO (b), percent predicted peak VO_2_ and changes in SV (c), peak VO_2_ and changes in CO (d), ΔVO_2_/ΔWR and changes in SV (e), ΔVO_2_/ΔWR and changes in CO (f), and ΔVO_2_/ΔWR and changes in CI (g). CI, cardiac index; CO, cardiac output; CPET, cardiopulmonary exercise testing; SV, stroke volume.

Next, we analyzed the association between the exercise capacity assessed by CPET or levels of BNP, and changes in hemodynamic parameters from before to after exercise. Changes in SV and CO were positively correlated to the level of peak VO_2_ (SV: *r* = 0.622 and *p* = 0.041, CO: *r* = 0.644, *p* = 0.033) (Figure 3a,b) and percent predicted peak VO_2_ (SV: *r* = 0.628 and *p* = 0.039, CO: *r* = 0.721, *p* = 0.012) (Figure 3c,d). In addition, changes in SV (0.603, *p* = 0.049) (Figure 3e), CO (*r* = 0.652, *p* = 0.03) (Figure 3f) and CI (*r* = 0.633, *p* = 0.036) (Figure 3g) were positively correlated with ΔVO_2_/ΔWR, a parameter reflecting the efficiency of oxygen uptake in response to work rate.

Furthermore, we compared parameters of CPET and BNP between groups with increased and decreased CO group during aerobic exercise (Table 3). The level of peak VO_2_ (16.9 ± 2.71 vs. 13.3 ± 1.11, *p* = 0.02) and ΔVO_2_/ΔWR (9.22 ± 1.11 vs. 6.28 ± 2.03, *p* = 0.01) were significantly higher in the increased CO group than the decreased CO group. The level of VO_2_ at AT tended to be higher in the increased CO group than the decreased CO group (11.9 ± 1.34 vs. 10.4 ± 0.69, *p* = 0.06); however, these changes did not reach statistical significance.

**TABLE 3:** Comparison of parameters of CPET and BNP between groups with increased and decreased CO group during aerobic exercise.

	Increased CO group (*n* = 6)	Decreased CO group (*n* = 5)	*p* value
BNP (pg/mL)	50.1 ± 33.4	67.1 ± 28.3	0.39
PeakVO2 (mL/kg/min)	16.9 ± 2.71	13.3 ± 1.11	0.02
VO2 at AT (mL/kg/min)	11.9 ± 1.34	10.4 ± 0.69	0.06
ΔVO2/ΔWR (mL/min/watt)	9.22 ± 1.11	6.28 ± 2.03	0.01
VE/VCO2 slope	30.3 ± 5.16	29.6 ± 8.55	0.87
Minimum VE/VCO2 (mL/mL)	29.4 ± 9.62	34.0 ± 4.93	0.36

*Note*: Continuous variables as mean ± SD.

Abbreviations: AT, aerobic threshold; BNP, brain natriuretic peptide; CO, cardiac output; CPET, cardiopulmonary exercise test; VE, minute ventilation; WR, work rate.

## 4 DISCUSSION

In this study, we demonstrated the use of non‐invasive measurement of hemodynamic parameters during CR. Our findings revealed a correlation between TFC both before and after aerobic exercise and the level of BNP. Additionally, we observed a correlation between changes in SV and CO during exercise and the level of peak VO_2_, as well as between changes in SV, CO, and CI during exercise and ΔVO_2_/ΔWR. Notably, we found that patients with increased CO during exercise exhibited greater values in peak VO2 and ΔVO2/ΔWR compared to those with decreased CO.

Right heart catheterization (RHC) remains the gold standard for measuring hemodynamic parameters among patients with cardiovascular diseases (Fincke et al., 2004). However, RHC is invasive and linked to complications such as bleeding or infection. To overcome these problems, non‐invasive devices for monitoring hemodynamic parameters have been developed. Thoracic electrical bioimpedance is an index of changes in the electric conductivity of blood flow which can be measured through skin, and enables non‐invasive calculation of CO (Kubicek et al., 1966). In the past few decades, this method has been modified and electrical velocimetry is a well refined recent algorithm (Bernstein & Lemmens, 2005). AESCULON mini is a real‐time monitoring device for hemodynamic status implementing the features of electrical velocimetry. It is already applied to clinical uses such as optimization of the treatment for acute heart failure with a patient suffered dilated cardiomyopathy (Nakayama, Iwama, et al. 2020), monitoring hemodynamic status in pediatric patients with single ventricle (Yoshitake et al., 2017) and monitoring CO during pediatric cardiac surgery (Tirotta et al., 2017). There are some reports which demonstrated the use of AESCULON during ergometer exercise echocardiography (Nakayama, Iwama, et al. 2020) and home‐based CR program (Chen et al., 2018); however, these reports did not demonstrate analysis of the relationship between exercise capacities and the level of BNP, and hemodynamic parameters obtained by AESCULON. Our results including the relationship between exercise capacity and the level of BNP, and hemodynamic parameters obtained by AESCULON, align with previous reports. TFC, a parameter for chest fluid volume, has a positive relationship to BNP (Muller, 2025). Confirming whether or not heart failure has worsened after exercise therapy is useful for confirming the appropriateness of the intensity of exercise therapy. Reduced peak VO2 in predominantly was attributable to limited increase in CO during exercise (Abudiab et al., 2013; Murabayashi et al., 1997). Peak VO2 is an important parameter of exercise capacity, related to the prognosis of the patients of heart failure (Sarullo et al., 2010). Furthermore, increase in peak VO2 is associated with a more favorable outcome in patients with chronic heart failure (Swank et al., 2012). Fluttering of ΔVO_2_/ΔWR was associated with reduced CO increase during CPET (Bandera et al., 2014). ΔVO_2_/ΔWR is one of the important parameters of exercise capacities as well as peak VO2. Decrease (Tanabe et al., 2002) and fluttering (Bandera et al., 2014) in ΔVO_2_/ΔWR reflects poor CO response to exercise and exacerbated cardiac reserve, respectively. Thus, hemodynamic monitoring using AESCULON during exercise training would be reliable method.

Furthermore, our findings imply that the use of AESCULON non‐invasive monitor would be utilized for clinical practice of exercise training program in certain situations. Patients with a history of myocardial infarction or cardiomyopathy are at high risk for developing heart failure; therefore, vigilant monitoring is essential to detect its onset or progression during CR program. In cases where symptoms such as dyspnea or peripheral edema are observed, body fluid retention could be immediately assessed by measuring TFC, which predicts BNP levels. This approach could facilitate confirming the safety of the exercise intensity during CR program and reconsideration of exercise intensity when the perceived exertion of the patient during exercise is lower or higher than usual. In addition, it would also be helpful for the early detection of new‐onset or worsening heart failure, or further clinical evaluation when the patient has a symptom related to worsening of heart failure such as shortness of breath or swelling of the foot.

When patients are unable to undergo CPET due to the patient's condition or a poorly equipped facility, aerobic exercise intensity is determined by heart rate (Brown et al., 2024), rating of perceived exertion (Borg, 1982) or talk test (Reed & Pipe, 2014). In such a situation, measurement of hemodynamic parameters using AESCULON would be a complementary method for the estimation of peak VO2 and ΔVO_2_/ΔWR.

On the other hand, there are several limitations to measurement with AESCULON. While reproducibility of AESCULON measurement is high (Trinkmann et al., 2016), compared with the pulmonary artery catheter thermodilution method, the AESCULON monitor provides less accurate absolute values of hemodynamic parameters, with a mean percentage error exceeding 30% (Tomaske et al., 2008). Higher height, gender of female, and higher CO are related to the inaccuracy of AESCULON measurement (Trinkmann et al., 2016). Placement of the electrodes also affects the inaccuracy of measurement (Sanders et al., 2020). We think that a baseline measurement of the parameters of cardiac function by the AESCULON mini would be required for each exercise session because the measurement of absolute values by AESCULON would be less accurate, and our result demonstrated the relevance between the exercise parameters obtained with cardiopulmonary exercise testing and not the absolute values of SV or CO but the changes in SV or CO.

As this study was a single‐center, there is a risk of patient selection. The sample was too small to reach definitive conclusions, and thus, the data are sufficient only to generate a hypothesis and the results should be interpreted with caution. A larger population is required to confirm our results. Additionally, the workload on the ergometer varied among patients, as exercise prescriptions were determined by the workload at AT evaluated by CPET. Furthermore, since the primary focus was on the feasibility of non‐invasive hemodynamic monitoring using AESCULON mini, this study lacks the parameters of ventilatory functions such as respiratory rate or tidal volume, which would affect cardiopulmonary function during exercise. Detailed ventilatory measurements will be important in future larger studies.

## 5 CONCLUSIONS

Some of the hemodynamic parameters obtained by a non‐invasive AESCULON monitor during aerobic exercise training were significantly associated with the level of BNP and exercise capacities. Non‐invasive hemodynamic monitoring using AESCULON would help us for the detection of the onset or worsening of heart failure and be a complementary method for the prediction of exercise capacities.

## AUTHOR CONTRIBUTIONS

Takayuki Fujiwara, Eisuke Amiya, and Masao Takahashi conceived and designed research. Takayuki Fujiwara, Yuto Konishi, Masao Takahashi, Kanako Hyodo, and Naoko Takayama contributed to data collection. Takayuki Fujiwara and Eisuke Amiya analyzed data. Takayuki Fujiwara wrote the manuscript. Eisuke Amiya, Issei Komuro, and Naoko Takayama revised the manuscript. All authors read and approved the manuscript.

## FUNDING INFORMATION

The authors have nothing to report.

## CONFLICT OF INTEREST STATEMENT

The authors declare no conflicts of interest.

## ETHICS STATEMENT

All components of standard informed consent, including the purpose of the study, risk, and benefits, were fully explained to the patients before enrollment. Written informed consent was obtained from each patient. The study protocol conformed to the tenets of the Declaration of Helsinki and was reviewed and approved by the Institutional Review Board of the University of Tokyo, Tokyo, Japan (10760).

## Data Availability

All data are available upon request.
